# An innovative method for manufacturing the Tuebingen palatal plate for infants with Robin sequence

**DOI:** 10.1186/s12887-020-2009-2

**Published:** 2020-03-04

**Authors:** Silvia Müller-Hagedorn, Jörg Arand, Thilo Scholz, Christian F. Poets, Cornelia Wiechers

**Affiliations:** 1 Interdisciplinary Center for Craniofacial Malformations, Tübingen University Hospital, Tübingen, Germany; 2 Department of Orthodontics, Tübingen University Hospital, Tübingen, Germany; 3 Department of Neonatology, Tübingen University Hospital, Calwerstrasse 7, D-72076 Tübingen, Germany

**Keywords:** Tuebingen palatal plate, Upper airway obstruction, Thermoplastic spur, Robin sequence, Glossoptosis, Orthodontics

## Abstract

**Background:**

Robin sequence (RS) is characterized by mandibular micro- and retrognathia, glossoptosis, upper airway obstruction and optionally a cleft palate. With an incidence of 1:8000, it belongs to the so-called rare diseases; 30–50% of patients have RS as part of a syndrome. A comparatively well-studied treatment option is the Tuebingen Palatal Plate (TPP), which has proven effective in both, isolated and syndromic RS, but often requires multiple endoscopies for perfect fit and effectiveness. We report on a new method for fitting the TPP with only one session of nasopharyngeal endoscopy resulting in the plate being finished in one day.

**Methods and results:**

First, a prototype is produced, consisting of a traditional acrylic palatal part and a velar extension made of thermoplastic resin, usually measuring 10x40mm. Using polymerization, a scale is added to the posterior part of the extension to help with determining its optimal length during endoscopic evaluation. The extension is pre-bent in the dental laboratory to achieve an approximate shape. During endoscopy, the prototype can be adjusted to the infant’s anatomy: first, the angulation is customized by controlled heating, bending and cooling of the thermoplastic spur. Second, the length of the spur is adapted by grinding its tip. Then the prototype is returned to the dental laboratory for completion; the final plate can be delivered to the patient on the same day. It acts by shifting the tongue into a more anterior position, thereby opening the airway and releasing upper airway obstruction, as well as by acting as a functional orthodontic appliance that stimulates mandibular growth through exerting pressure on the base of the tongue.

**Conclusions:**

With the thermoplastic spur presented here, a TPP can be produced within one day, requiring only one endoscopy. This approach may facilitate fabricating the TPP.

## Background

Robin sequence (RS) is characterized by micro-, retrognathia, glossoptosis and optionally a cleft palate. It presents clinically with intermittent upper airway obstruction (UAO) and feeding difficulties, which are most severe during the first postnatal months. A recent epidemiological study determined a birth prevalence of 12.4 per 100,000 live births [1].

RS may occur as an isolated entity (isolated RS; iRS), as a component of a syndrome (syndromic RS; sRS) or in association with other malformations (associated RS; aRS). In a recent study, 40% of RS were isolated and 60% syndromic or associated with other malformations [2]. More than 50 syndromes have been described in association with RS, the most common being Stickler, Nager, Treacher Collins and velocardiofacial syndrome [3].

Four types of UAO can be determined endoscopically according to the Sher classification [4]. The so-called type I obstruction, i.e. true glossoptosis, is found in 90% of patients with iRS, but in 44% of those with syndromic or associated RS [5]. In a type II obstruction the velum is compressed by the tongue to the posterior pharyngeal wall, and type III and IV obstructions are caused by a lateral or circular narrowing of the pharyngeal wall.

Treatment of RS is indicated mainly in case of upper airway obstruction, i.e. a mixed obstructive apnea index (MOAI) > 3/h, indicating the presence of > 3 mixed or obstructive apneas per hour in a sleep study, or other symptoms of UAO such as respiratory noise, snoring, sternal retractions, sweating during feeds, feeding difficulties and failure to thrive. Treatment often involves invasive procedures, such as mandibular distraction osteogenesis, tongue-lip adhesion or mandibular traction, or interventions that bridge the narrow airway, e.g. nasopharyngeal airway, tracheostomy or application of continuous positive airway pressure (CPAP). Mild cases are often treated by prone positioning, but this is fraught with an increased risk of sudden infant death syndrome [6]. In Germany, according to a recent survey [7], 37% of infants with RS are treated with a pre-epiglottic baton plate that is comparable to the Tuebingen Palatal Plate (TPP) and is a less invasive, yet effective and comparatively well studied alternative for patients with isolated [8–11] and syndromic RS [12]. This treatment consists of the standard or modified TPP in combination with Manual Orofacial Therapy according to Castillo Morales [13] and Brondo [14]. Up to now, there is no protocol available for manufacturing this plate in a standard way.

The TPP shifts the tongue into a more anterior position and erects the epiglottis, thereby widening the posterior airway and releasing UAO. Two parameters are important to achieve this, namely the angulation and the length of the spur. Previously, it was often laborious to fit the TPP, as several endoscopies were necessary to optimize both parameters. For each modification of the angulation of the spur, the prototype had to be returned to the dental laboratory; afterwards a repeat endoscopy usually became necessary.

Here, we present a new way of fitting the TPP that requires only one session of endoscopy so that the plate can be produced in one day. This is important from a patients’ point of view, because their upper airway obstruction may be released faster, feeding training started earlier and children discharged home sooner.

## Methods

### Preliminary procedures

Infants, ideally neonates, have a maxillary imprint taken using a custom-made impression tray and alginate (Tetrachrom-Super-Alginat, ISO 1563, Klasse B, Typ I, Kaniedenta, Herford, Germany) or a-silicone (Freealgin, Zhermack Dental, Badia Polesine, Italy).

This imprint has to cover the entire hard palate, the alveolar ridges and the vestibule. The procedure is carried out in the neonatal intermediate care unit under cardiorespiratory monitoring, in the presence of an experienced neonatologist and with a nasopharyngeal airway in place to secure the airway. Then a plaster cast is produced using high precision dental plaster (Girodur Type IV, Synthetic Superhard Stone Plaster for Sectioned and Master Models DIN EN 26873, white, Girrbach Dental GmbH, Pforzheim, Germany). Using this cast, appliances are produced. A velar extension of approximately 4 cm in length is added to the plate representing the spur.

## Results

### Manufacturing of the TPP

#### Classical method

The shape of the TPP’s velar extension is modelled from dental wax and attached dorsally to the plaster cast [12]. Its inclination or angle is chosen so that it is positioned adjacent to the dorsum of the tongue and shifts the tongue into a more anterior position, thereby widening the posterior airway space. Up to now, this depends on individual experience as no standard values have yet been published for patients with RS. The whole appliance is made from acrylic (autopolymerizing methylmethacralate, Orthocryl®, Dentaurum, Pforzheim, Germany) with the spur having a dark color to facilitate its recognition during endoscopy. During the first endoscopy session, the type of obstruction is assessed and the prototype evaluated, but the shape of the prototype is only estimated, so that the plate often has to be returned to the dental laboratory to individualize the extension by adding methylmethacrylate on one side and removing material on the opposite side to change the inclination of the spur. In an additional endoscopy, the angulation and the length of the individualized spur are assessed again, because the length depends on the inclination.

#### Innovative method

**Fig. 1:**
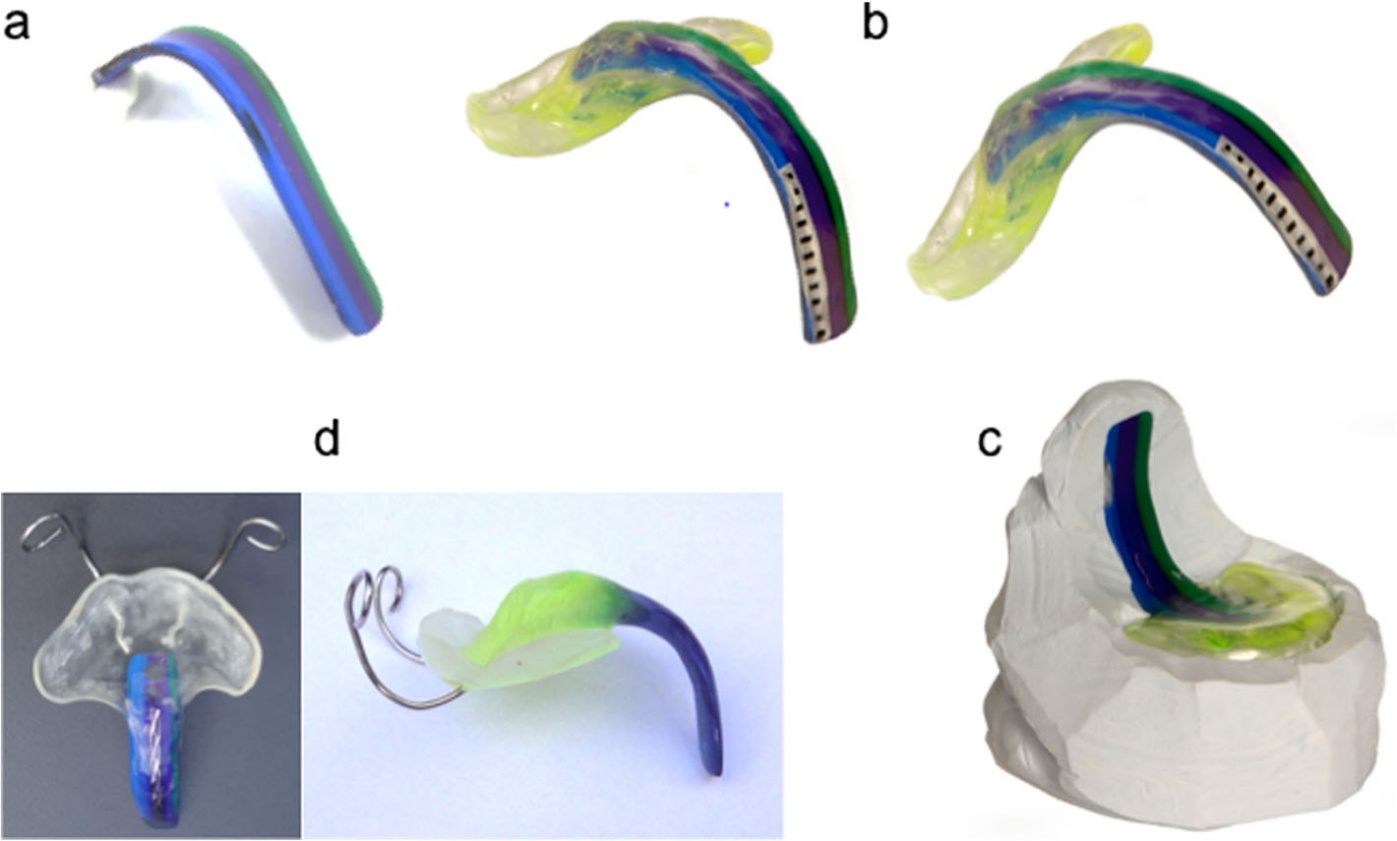
Production of the Tuebingen Palatal Plate with velar extension (clockwise from top left): **a**. Pre-bent spur. **b**. Prototype with thermoplastic spur and scale. **c**. Imprint of prototype (serves as mould for final TPP). **d**. Final Tuebingen Palatal Plate with extraoral wires attached

With the suggested innovative method, the palatal part remains the same, but the extension is now made from thermoplastic resin: A multicolor striped thermoplastic material (Biocryl M®, hard elastic, monomer free plates of laminated copolymer, bond to acrylic, Scheu Dental, Iserlohn, Germany) is used, cut into a 3-colored strip (purple, green and blue) measuring 10 × 50 mm, which is then heated and bent according to the dental practitioner‘s experience and with the help of a plaster mould (Fig. 1 a). Then, the palatal part and the extension are connected by polymerization with methylmethacrylate (Orthocryl®) to get a prototype (Fig. 1 b). The 3 colors allow distinguishing between left and right during endoscopic evaluation. A scale is added by polymerization with a transparent resin of methylmethacylate (Orthocryl®) to the posterior part of the extension to adjust its length during endoscopy in an easier and more precise way (Fig. 1 b).

**Fig. 2:**
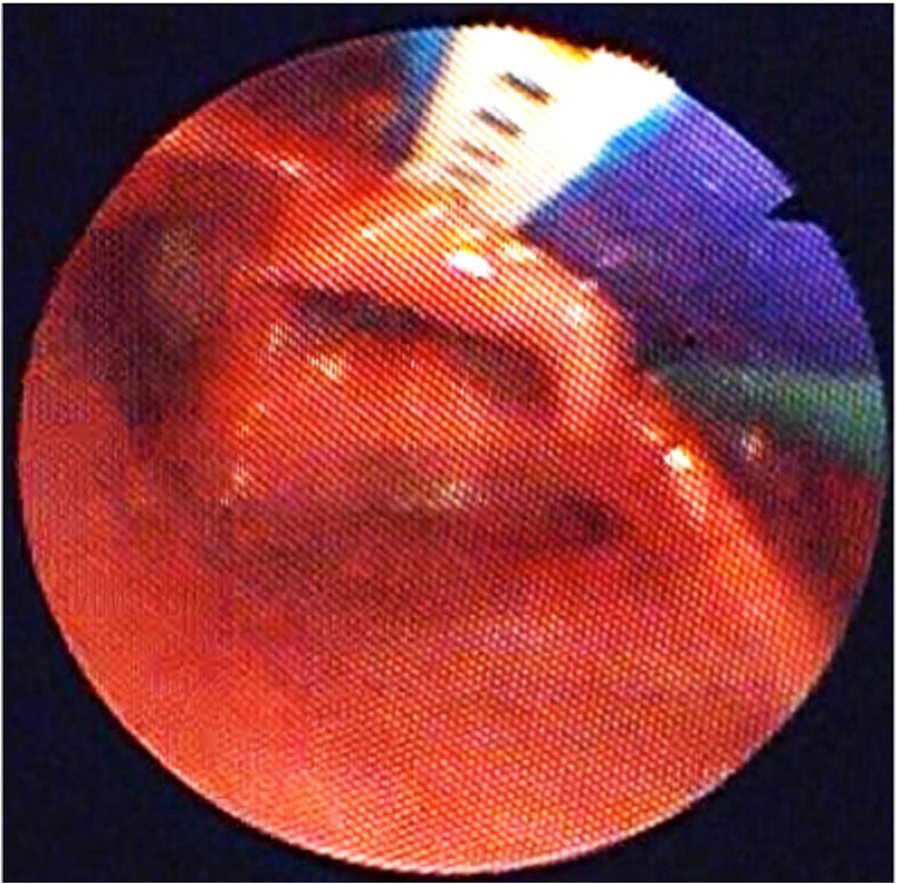
Nasopharyngeal endoscopy with prototype in situ. Here, the spur is not steep enough and still too long

The latter helps to fit the length of the velar extension to the infant’s anatomy (Fig. 2). The ideal angle of the velar extension can be achieved by controlled heating, bending and cooling of the thermoplastic extension. The ideal length is reached by grinding the tip of the extension. The fit of the revised shape of the extension is then controlled during the same endoscopy. Once the prototype has shown its effectiveness during endoscopy (anatomically and clinically), it is returned to the dental laboratory for finishing.

### Nasopharyngeal endoscopy

In general, first of all the type of obstruction according to the Sher classification [4] is defined and then, in a first step of customization, the angulation of the extension is adjusted and its length approximated. In a second step the correct length of the extension is determined more precisely, because the angulation also affects the length of the spur.

The extension has to shift the tongue into a more anterior position, thereby widening the pharyngeal airway. The root of the tongue has to be uploaded and the epiglottis erected. Only minimal pressure on the tongue in anterior direction is used to prevent it from bulging laterally of the spur. If the inclination of the spur is too steep, the plate cannot be incorporated (tactile impression) and the infant develops difficulty swallowing; this also carries a risk of impression marks. Concerning length, the tip of the spur is ideally located just above the epiglottis. Complete mobility of the epiglottis has to be ensured.

During endoscopy, which is carried out without any sedation, it is important that the infant lies straight on its back and that the cervical spinal column is not in hyperextension, otherwise adjustment of the spur may not be correct. Once the spur seems to be in a good position, the infant’s head is turned left and right to check the spur’s position during head movements.

### Completion of the TPP

After having shown its correct fit during endoscopy, the TPP is returned to the dental laboratory for completion. There are 3 options for producing the final TPP:

1. An imprint of the whole prototype is done with c-silicone (HS-Knetsilicon 2 Technik Eurosil Max 2 Lab /HS-C-Silikon 2 Härter Eurosil Max 2 Catalyst Paste, Henry Schein Dental, Langen, Germany) (Fig. 1 c). With this mould a complete final TPP is produced from methylmethacrylate (Orthocryl®). The spur, again, is in a dark color, so that it can easily be identified during endoscopy and the palatal part is in a bright transparent color (Fig. 1 d). The advantage of this method is that the original prototype can be re-used for further endoscopy (stepwise procedure in severe cases).
2. After having produced the above mould, the spur of the prototype is split off and the palatal part replaced in the mould. Then, only the spur is added to the palatal part with the help of the mould, again using dark colored methylmethacrylate (Orthocryl®) (Fig. 1 d). The advantage of this method is that it saves time in the dental laboratory, but the prototype is lost.
3. The whole prototype is used for the final TPP and only the spur embedded in transparent methylmethacrylate (Orthocryl®). Here again the prototype is lost, but this is the most time-saving method. The advantage of this method is that it makes use of the striped spur, so that the laterality of potential pharyngeal impression marks can easily be identified (Fig. 1 d).

In a type I obstruction according to Sher [4], as often identified in isolated RS, a simple spur is sufficient. In types II to IV, a ring, short tube or an artificial airway have to be added to the spur [12]. In all cases, however, correct adaptation of the spur is mandatory as a first step. In a second step, additional applications (ring, tube) can be added to the standard TPP. Then, the endoscope is introduced into the tube or artificial airway of the plate and evaluation is done by inserting the plate together with the endoscope, offering a view on the airway. If the latter appears clinically and endoscopically open, the plate is accomplished.

Then, a security wire is incorporated into the extension to safeguard the device against mechanical failure. Furthermore, extraoral wires are added to improve retention of the plate and to counteract the force from the base of the tongue. After this step the TPP can be delivered to the patient.

### Use of the TPP

In infants and during the first 3 months of treatment plates are worn continuously and only removed briefly for cleaning purposes once a day. Involving parents in handling the plate as early as possible is important for treatment success, as they have to handle, clean and insert the plate once home, as well as know how to inspect the gingiva for pressure marks and how to feed their baby. In general, patients are discharged from the neonatal intensive care unit around 3 weeks after treatment onset, but only if parents feel confident with their above tasks.

A follow-up visit including a sleep study is planned approximately 3 months after hospital discharge, which coincides with the time a new plate becomes necessary due to craniofacial growth. If the plate becomes too small, a notch appears on the alveolar ridge or breathing becomes labored again. Palatal plate treatment is discontinued once infants show a normal tongue position and mandibular size (normal profile), usually after around 6 months of treatment. For a new plate, again an imprint is taken, a prototype produced and adjusted by nasopharyngeal endoscopy and a final TPP fabricated to be evaluated again by a sleep study.

## Discussion

The effects of this treatment should be discussed on several levels: medical, economic, material used for the spur, procedure and applicability.

### Effects on a medical level

RS is characterized by micro- and retrognathia, glossoptosis and cleft palate, the latter being present in about 80% of cases. Infants with RS suffer from intermittent UAO, feeding difficulties and failure to thrive.

The TPP addresses particularly glossoptosis as the major anomaly in RS, as it prevents the tongue from falling back into the pharynx, shifts the tongue into a more anterior position and erects the epiglottis, thereby opening the airway (immediate effect). The TPP also seems to provide a stimulus for mandibular growth, either because it shifts not only the tongue but also the mandible into a more anterior position [15], or because it allows for normal sucking and swallowing. The more anterior mandibular position may stimulate condylar growth, which enables skeletal adaptation [16]. This results in catch-up growth of the hypoplastic mandible (long-term effect). Therefore, the TPP can be considered as a functional orthodontic appliance. As mentioned above, the spur results in the tongue to achieve a more physiologic and horizontal position, allowing for sucking and swallowing. Therefore, it can be considered as a curative treatment in contrast to most other RS treatment options. Thus, UAO and feeding difficulties can be treated and failure to thrive prevented. These are important goals. Furthermore, it is a less invasive treatment option helping to avoid more invasive, mostly surgical, treatments.

Now, with the thermoplastic spur, the TPP can be delivered in one day and patients can be spared at least one session of nasopharyngeal endoscopy. All in all, treatment, including functional and feeding training, can be started one day earlier with the protocol reported here than with the classical way of customizing a TPP.

Acceptance of the TPP by patients is easier the earlier treatment is started. The optimal age for onset of treatment is in the neonatal period. In general, the plate has to be worn for approximately six months, until the patient has achieved a normal facial profile and tongue function. Furthermore, a normal sleep study (i.e., MOAI< 1) after the plate has not been worn for 10–14 days is required. For this reason it is important that a larger number of neonatal and orthodontic departments can offer this kind of treatment. No severe side effects could be observed except for impression marks, which could be treated by grinding and polishing the plate, and occasionally skin rashes caused by the adhesive tape used to fixate the extra-oral bows.

### Effects on an economic level

With the treatment option presented here, the TPP can be fitted in one day and with one session of nasopharyngeal endoscopy. Prior to introducing the thermoplastic spur, 2 or more endoscopies were often needed and the prototype had to be returned at least once to the dental laboratory for modifying the spur, resulting in more working hours for the dental technician. As the new procedure can be completed faster than with the classical method, discharge from hospital may occur earlier, resulting in cost savings.

### Material used for the spur

Biocryl® M, a hard elastic thermoplastic material developed for pressure molding technique in orthodontics, was used for the prototype spur (Scheu Dental, Iserlohn, Germany) with 2 mm thickness. Chemically, it is a laminated copolymer (polyvinylchloride), free of monomer and bond to acrylic is possible. The material has been tested on biocompatibility according to DIN EN ISO 10993. It meets the requirements for biological compatibility for medical products [17]. This practice implies off-label use of this material. However, we wish to stress the above characteristics and our positive experience with this approach in at least 30 patients. Nonetheless, some precautions have to be taken: the thermoplastic spur must have a width of at least 10 mm and 2 mm thickness to ensure its stability, and only a discrete spur deformation should occur with tongue movements during endoscopy. A further increase in the spur’s stability is achieved by adding a scale to the posterior and distal part of the spur and by coating it with a transparent resin of polymethylmethacrylate. However, there may still remain a discrete discrepancy between the angulation of the prototype and the final TPP due to some remaining elasticity of the thermoplastic spur, but we this was found to be clinically negligible. Finally, we warn against bending the spur during adaptation without sufficient prior heating to prevent fracture of the material. Also, it is important that the spur is well pre-bent before first insertion.

### Procedure

Using an alginate for the impression has the advantage that it hardens in less than 1 min, but the disadvantage that it may rupture due to its low consistency, so that some parts of the imprint may remain in the cleft. An alternative impression material is a hydrophilic addition silicone (a-silicone). An advantage of a-silicone is that there is no risk of impression material tearing off into the cleft, but it takes longer to set than an alginate and thus carries an increased risk of respiratory problems and of a vasovagal response.

Recently, intraoral scans have become feasible. An intraoral digital impression technique for neonates with bilateral cleft lip and palate by intraoral scanning has been described. The direct oral scan can be exported as stereolithographic files and then be printed. Digital scanning has been described as fast, accurate, and safe if compared to a conventional alginate impression technique [18].

It has to be added that conventional impression taking in an infant is very technique-sensitive and always carries the risk of aspiration and airway obstruction. Therefore, appropriate precautions must be taken during the procedure. In the future, digital workflow may gain an important role and may supersede conventional impression taking.

For endoscopic adjustment the pediatrician should be familiar with nasopharyngeal endoscopy in order to complete the procedure quickly in the unsedated infant.

Adjustment of the plate depends on the experience of the practitioner. In general, if the plate can only be incorporated with some resistance, the spur is too steep. The plate should slide quite easily into its final position. If the spur is too steep, patients will not tolerate the TPP and develop difficulty swallowing. This also carries a risk of developing impression marks.

For adjustment of the TPP the endoscopic evaluation of the airway with the plate in situ is as important as the clinical impression of the patient. No respiratory noises or snoring should be noticeable with the plate in situ. A quiet sleep, without hyperextension of the cervical spinal column and no signs of sweating are good indicators for a properly functioning plate. After 48 h of uneventful treatment, effectiveness of the TPP should be verified by a sleep study. In case of persisting UAO the plate has to be modified.

### Limitations and outlook

The most important limitation of our new approach to TPP production reported here is that it requires a neonatal care unit and an interdisciplinary team consisting of neonatologists trained in nasopharyngeal endoscopy, orthodontists working together with experienced dental technicians, pediatric sleep specialists and speech therapists familiar with orofacial regulation therapy. An experienced nursing team and regular feeding training are also of great importance, the former also to train the parents in handling the plate.

TPP treatment has been accused of being difficult to apply. We present an easy way to fabricate the TPP. By this treatment protocol only one session of endoscopy is necessary. In case of isolated RS the spur can be bent into an optimal position at the first endoscopic evaluation. The estimated time for an endoscopy session for adaptation of the spur in length and angulation, with interruptions, is about 10–15 min.

For inexperienced users we recommend to preserve the prototype, because in case of insufficient UAO treatment, identified through a sleep study or clinical observation, it can be used for further endosopic evaluation using a spur with another inclination or length. The spur of the original prototype can be easily modified by heating and bending.

## Conclusion

We consider the method described here sufficiently instructive for manufacturing the TPP. This treatment option is only minimally invasive yet potentially curative. It should thus enable clinicians to offer this kind of treatment to infants with RS even with teams less experienced with this treatment modality. Furthermore, it is more cost-effective than the traditional method of producing the TPP.

## Data Availability

Not Applicable.

## Abbreviations

aRS: Associated Robin sequence
a-silicone: Addition silicone
CPAP: Continuous positive airway pressure
c-silicone: Condensation silicone
iRS: Isolated Robin sequence
MOAI: Mixed obstructive apnea index
RS: Robin sequence
sRS: Syndromic Robin sequence
TPP: Tuebingen Palatal Plate
UAO: Upper airway obstruction
